# Verbal working memory and processing speed: Correlations with the severity of attention deficit and emotional dysregulation in adult ADHD

**DOI:** 10.1111/jnp.12260

**Published:** 2021-07-04

**Authors:** Espen Anker, Geir Ogrim, Trond Heir

**Affiliations:** ^1^ Oslo ADHD Clinic Norway; ^2^ Østfold Hospital Trust Norway; ^3^ Gillberg Neuropsychiatry Centre University of Gothenburg Sweden; ^4^ Institute of clinical Medicine University of Oslo Norway

**Keywords:** Adult ADHD Self‐Report Scale, Attention Deficit Hyperactivity Disorder, emotional dysregulation, processing speed, Wechsler Adult Intelligence Scale, working memory

## Abstract

**Objectives:**

The Diagnostic and Statistical Manual of Mental Disorders, Fifth Edition (DSM‐5), emphasizes symptoms severity with regard to the diagnosis of attention deficit hyperactivity disorder (ADHD). Many clinicians use neuropsychological test results as objective measures of cognitive functions as part of the diagnostic work‐up. The aim of this study was to investigate whether the psychometric test results regarding verbal working memory and processing speed are useful as indicators of the severity of attention deficits and emotional dysregulation in adults with ADHD.

**Methods:**

This observational cross‐sectional clinical study included 418 adults diagnosed with ADHD according to the DSM‐5. Attention deficit severity was defined based on the inattentive subscale of the Adult ADHD Self‐Report Scale. Emotional dysregulation was assessed with the Deficient Emotional Self‐Regulation scale. Verbal working memory was measured with the Working Memory Index (WMI), and processing speed was measured with the Processing Speed Index (PSI) from the Wechsler Adult Intelligence Scale, third edition.

**Results:**

The full‐scale intelligence quotients of the participants were in the normal range, with expected reductions in verbal working memory and processing speed. Only processing speed was associated with attention deficits (β = −.056, *p* = .003). The association between the psychometric test result for verbal working memory and processing speed and that between the severity of attention deficits and emotional dysregulation were weak (R^2^ < .1) and mostly non‐significant.

**Conclusion:**

The psychometric index scores for verbal working memory (WMI) and processing speed (PSI) seem to have limited utility as indicators of the severity of attention deficits and emotional dysregulation in adult ADHD patients.

## Background

When assessing attention deficit hyperactivity disorder (ADHD), clinicians often use results from neuropsychological tests to obtain objective measures of the patient’s cognitive function to supplement clinical assessments that are based on subjective self‐report questionnaires and interviews. There are several reasons for this. The symptoms constituting ADHD are extreme variants of normal behaviours, and the boundary between normality and pathology is based on clinical judgement. Several studies have shown that the diagnosis of ADHD is highly dependent on the source of the report, thus underscoring the need for objective assessments (Barkley, Fischer, Smallish, & Fletcher, 2002). In addition, some symptoms of ADHD may reflect the presence of other disorders. Clinical difficulties related to attention deficits have been confirmed in several studies involving adults with ADHD (Barkley, 2010a; Bush, 2010; Halleland, Haavik, & Lundervold, 2012; Seidman, 2006), and they have been reported in studies assessing the severity of nine Diagnostic and Statistical Manual of Mental Disorders (DSM)‐defined inattentive symptoms, for example those reflected in the Adult ADHD Self‐Report Scale (ASRS) inattentive subscore (Fredriksen et al., 2014; Kessler et al., 2005; Silverstein et al., 2019). Adults with ADHD also have impaired executive functioning (Silverstein et al., 2020), but they do not necessarily exhibit attention deficits on psychometric tests (Fabio & Caprì, 2017).

Emotional dysregulation, which is recognized as a transdiagnostic factor (Aldao, Gee, De Los, & Seager, 2016), is a common feature in ADHD even though it is not part of the criteria for the disorder (Barkley, 2010b; Barkley & Fischer, 2010; Connor, Steeber, & McBurnett, 2010; Hirsch, Chavanon, Reichmann, & Christiansen, 2018; Landaas, Halmøy, Oedegaard, Fasmer, & Haavik, 2012; Retz, Stieglitz, Corbisiero, Retz‐Junginger, & Rösler, 2012; Shaw, Stringaris, Nigg, & Leibenluft, 2014; Surman et al., 2011, 2013). The clinical expression of emotional dysregulation is observable as excessive and inappropriate emotional expressions, irritability, and outbursts of temper (Shaw et al., 2014; Stringaris, 2011). This impulsivity with regard to anger is an important and disabling feature associated with ADHD (Bunford, Evans, & Langberg, 2018; Skirrow & Asherson, 2013). There is a strong relationship between anger and cognitive distortions (Chereji, Pintea, & David, 2012), and frequencies of both the expression of anger and cognitive distortion have been found to be associated with impaired executive functioning (Persampiere, Poole, & Murphy, 2014). Psychometric testing for executive functioning is frequently used in the neuropsychiatric assessment of ADHD, and one core executive function is verbal working memory, which involves the mental retention of auditory information that enables future action (Diamond, 2013). In several studies, a close relationship was found between emotional regulation and the level of working memory (Barkley, 1997; Jasielska et al., 2015; Jensen et al., 2018; Lima, Peckham, & Johnson, 2018; Rutherford, Booth, Crowley, & Mayes, 2016). Emotional dysregulation has not yet been conclusively defined, and there are several ways to measure, interpret, and categorize emotional dysregulation. In our study, emotional dysregulation was assessed based on eight relevant items (see Table 5) from the larger Current Behaviour Scale–Self‐Report, known as the Deficient Emotional Self‐Regulation (DESR) questionnaire (Barkley, 1997, 2010a; Biederman et al., 2012; Surman et al., 2013). The concept of emotional dysregulation has different aspects, involving both strong emotional responses and the impaired regulation of emotions.

Working memory is an important executive function (Diamond, 2013), and working memory deficits are well documented in adults with ADHD (Alderson, Kasper, Hudec, & Patros, 2013). Psychometric tests of working memory can be used to differentiate between those with and without ADHD in both children and adults (Willcutt, Doyle, Nigg, Faraone, & Pennington, 2015). According to Allan Baddeley, working memory consists of a central executive and two content/modality‐specific components: a phonological loop that handles verbal content and a visuospatial sketchpad that handles spatial content (Baddeley & Logie, 1999). Attention deficits/attention control is strongly related to central executive function. In addition, attention deficits in ADHD seem to have a stronger relation to the visuospatial component than to the phonological loop (Faraone & Biederman, 2005; Nigg et al., 2005; Woods, Lovejoy, & Ball, 2002). Different theoretical models have been developed to attempt to gain insight into working memory deficits in ADHD (Barkley, 1997; Diamond, 2005; Rapport, Chung, Shore, & Isaacs, 2001). In our sample, we analysed whether verbal working memory was correlated with the severity of either attention deficits or emotional dysregulation in ADHD.

Psychometric tests of processing speed measure the efficiency of cognitive executive skills, and impairments revealed by such tests have been linked to reports of attention deficits in children with ADHD (Kibby, Vadnais, & Jagger‐Rickels, 2019; Thorsen, Meza, Hinshaw, & Lundervold, 2018) and adults with ADHD (Tucha et al., 2017). Impaired processing speed explains to a large extent the impairment in executive functions (Butzbach et al., 2019). Impaired processing speed is linked to inattentive behaviour in children with ADHD (Kubo et al., 2018), and slow processing speed predicts social functioning in ADHD patients with inattention (Thorsen et al., 2018). It seems that slower processing speed and attention deficit coexist and are important predictors of academic achievement (Mayes & Calhoun, 2007). Studies have shown that the WAIS test results can be used to adequately discriminate between adults with and without ADHD, although the intragroup heterogeneity is substantial (Frazier, Demaree, & Youngstrom, 2004; Thaler, Bello, & Etcoff, 2013; Theiling & Petermann, 2014; Woods et al., 2002). Measures of working memory and processing speed can be used to discriminate adequately between ADHD and control groups (Nikolas, Marshall, & Hoelzle, 2019). Deficits in verbal working memory have been reported to be associated with parent‐rated emotional dysregulation in children with ADHD (Bunford et al., 2018), but others have not found this association in adults with ADHD (Chereji et al., 2012; Skirrow & Asherson, 2013). In our sample, we measured the correlations between processing speed and the severity of attention deficits and emotional dysregulation in adult ADHD patients. The severity of the symptoms of ADHD is emphasized in the DSM‐5 (American Psychiatric Association, 2013), but whether the WAIS test scores are useful as an indicator of the severity of attention deficits and emotional dysregulation is unknown.

We hypothesize that verbal working memory and processing speed are correlated with the severity of attention deficits and emotional dysregulation, even after controlling for covariates. The covariates that may affect the associations between neurocognitive test results and the clinical features of ADHD include age (Ardila, 2007), sex (Rucklidge & Tannock, 2001), education level (Reitan & Wolfson, 1995), and depression (Gorlyn et al., 2006).

The aim of this study was to determine whether the WAIS test results for verbal working memory and processing speed are useful as indicators of the severity of (1) attention deficits and (2) emotional dysregulation in adults with ADHD.

To broaden our understanding of the topic, we also sought to determine whether each item on the attention deficit and emotional dysregulation scales was correlated with the psychometric test results for verbal working memory and processing speed.

## Material and methods

This was an observational cross‐sectional clinical study.

### Participants

The study sample consisted of 418 adults aged 18–65 years who fulfilled the criteria for a diagnosis of attention deficit hyperactivity disorder (ADHD) according to the DSM, Fifth Edition (DSM‐5; American Psychiatric Association, 2013). They were referred to a private psychiatric outpatient clinic in Oslo, Norway, that specializes in psychiatric examination, assessment, and treatment of adults with ADHD.

Recruitment was conducted between 2014 and 2018. All patients participating in the study were assessed by a psychiatrist with the semi‐structured Diagnostic Interview for ADHD in Adults, second edition (DIVA 2.0; Kooij & Francken, 2010). The DIVA 2.0 is a reliable tool for assessing and diagnosing adult ADHD (Ramos‐Quiroga et al., 2019). A clinical diagnosis of ADHD was made according to DSM‐5 (American Psychiatric Association, 2013). From 2014 to 2018, 418 of the assessed patients fulfilled the diagnostic criteria for ADHD and were invited to participate in the study. Sixty‐five per cent of them were self‐referred, and thirty‐five per cent had been referred by health care practitioners. All 418 patients (100%) gave their written consent to participate and were included in the study. In accordance with the diagnostic criteria, all participants had ADHD symptoms before the age of 12. At the time of inclusion in study, they had severe ADHD symptoms that resulted in marked impairments in social or occupational functioning. There were no exclusion criteria. None of the participants were under the influence of stimulant medications, alcohol, or other drugs during the clinical assessment or administration of the WAIS test. Comorbid disorders were assessed using the Mini‐International Neuropsychiatric Interview (MINI).

The study was approved by the Regional Medical Ethics Committee, South‐East Norway 2015/426. Assessments and the handling of data were carried out in accordance with the relevant ethics standards and the principles of the Declaration of Helsinki.

### Measures

The age of the participants was recorded in years at the time of enrolment. Sex was recorded as female (scored as 0) or male (scored as 1) based on patient self‐report. The following sociodemographic information was collected. Marriage or cohabitation was scored as 1, and all other statuses were scored as 0. If the participant was living with children of whom they had at least partial custody, a score of 1 was recorded; otherwise, even if they had no children, or children who lived somewhere else, a score of 0 was recorded. Education level was categorized by the number of years of education as follows: 12 years or fewer was scored as 1, 13–15 years was scored as 2, and more than 15 years was scored as 3. Employment was defined as ‘yes’ and scored as 1 if that employment was reported as the main source of income; otherwise, it was scored as 0.

The level of depression was measured by the Montgomery–Åsberg depression rating scale (MADRS), with all ten items summarized as a total score ranging from 0 to 60 (Montgomery & Åsberg, 1979). The MADRS is a reliable and valid screening instrument for the evaluation of depression (Davidson, Turnbull, Strickland, Miller, & Graves, 1986).

ADHD symptom severity was measured using the Adult ADHD Self‐Report Scale (ASRS) Symptom Check List, v1.1 produced by the World Health Organization (World Health Organization, 2003). The ASRS is a reliable and valid screening instrument for the evaluation of ADHD in adults (Fredriksen et al., 2014; Kessler et al., 2005; Silverstein et al., 2019). The ASRS questionnaire is composed of 9 attention deficit items (items 1–4 and 7–11) and 9 hyperactive‐impulsive items (items 5, 6, and 12–18; Fredriksen et al., 2014). Response options for each item range from 0 to 4. The items were marked by the participant as never (0), rarely (1), sometimes (2), often (3), and very often (4). This yielded a total attention deficit score ranging from 0 to 36 (see Table 4).

Emotional dysregulation was assessed with a questionnaire containing eight items from the 99 items on the Current Behaviour Scale–Self‐Report (CBS‐SR) questionnaire, known as the DESR scale (Barkley, 1997, 2010b; Barkley & Fischer, 2010; Biederman et al., 2008; Surman et al., 2013). The eight items were as follows: 1: Quick to get angry or become upset; 2: Easily frustrated; 3: Overreact emotionally; 4: Easily excited by activities going on around me; 5: Lose my temper; 6: Argue with others; 7: Am touchy or easily annoyed by others; and 8: Am angry or resentful. The eight items are described in Table 5, and their response options range from 0 to 3. The items were marked by the participant as never or rarely (0), sometimes (1), often (2), and very often (3). This yielded a total ED score ranging from 0 to 24 (see Table 5).

Verbal working memory was assessed by the Working Memory Index (WMI; tests of Arithmetic, Digit Span and Letter‐Number Sequencing), and processing speed was assessed with the Processing Speed Index (PSI; tests of Digit Symbol‐Coding, Symbol Search) from the Wechsler Adult Intelligence Scale, 3rd edition (WAIS‐III).( Evers et al., 2012; Kaufman, 1999; Wechsler, 1997; Wechsler, Nyman, & Nordvik, 2003) The test scores included in the statistical analyses were the age‐corrected scale scores.

### Procedure

The data were collected during routine assessments performed by a psychiatrist in an outpatient clinic. Psychometric testing was completed by a special education teacher with experience and expertise in ADHD and the use of the WAIS‐III.

After the assessment, the patients were asked if they approved the use of their clinical information in an anonymous form in the statistical analyses for this clinical trial.

### Statistical analysis

Frequencies with per cent proportions are reported for all categorical variables and means with standard deviations are reported for continuous descriptive variables. We performed chi‐square tests or *t*‐tests to compare sociodemographic characteristics between females and males. All tests were two‐tailed, and differences were considered significant if *p* < .05. Continuity correction was performed, and Asymp. Sig. (2‐sided) was recorded. *t* Tests were used to compare continuous variables between females and males.

We used linear regression analyses to examine associations between measures of self‐reported attention deficit (the ASRS subscore), or self‐reported emotional dysregulation (the DESR scale) as the dependent/outcome variables, and sociodemographic variables, the MADRS score, the Working Memory Index (WMI) and the Processing Speed Index (PSI) as the independent variables. The test scores from the WAIS‐III included in the statistical analyses were the age‐corrected scale scores. We used Cronbach’s alpha to assess the internal consistency and reliability of the eight items from the DESR scale. Cronbach’s alpha for the eight scale items in our sample was .86, indicating high internal consistency.

Given our two hypotheses, we used the Bonferroni correction and considered differences significant if *p* was < .025. For all association tests, beta ratios with 95% confidence intervals were calculated as the measurement of the effect size. To measure the explained variance, we used the *R* ^2^‐squared value. If the *R* ^2^ value was <.30, the effect was considered weak. If the R^2^ value was between .30 and .50, the effect was considered moderate. If the R^2^ value was >.50, the effect was considered strong (Moore, Notz, & Notz, 2006).

We used Pearson correlation analyses to examine the associations of each of the nine attention deficit items on the ASRS and each of the eight emotional dysregulation items on the DESR scale with the Working Memory Index (WMI) and the Processing Speed Index (PSI).

There were no missing data. All statistical analyses were performed using IBM SPSS version 22 (IBM, 2013).

## Results

Table 1 shows the demographics, depression severity (as the MADRS score), attention deficit ASRS subscale score, hyperactive‐impulsive ASRS subscale score, total WAIS score, and four WAIS subindex scores in the females (*n* = 188) and males (*n* = 230) in this study. More females than males lived with children. A total of 61.7% of the participants reported employment as their main source of income, and nearly 51.4% reported that they had received more than 12 years of education. The frequencies of psychiatric comorbidities in this sample were 12.9% for depressive disorders, 31.8% for anxiety disorders, and 13.6% for substance use disorders.

**Table 1:** Demographic characteristics, depression (MADRS score), ASRS scores measuring attention deficits and hyperactivity‐impulsivity separately, emotional dysregulation measured by the Deficient Emotional Self‐Regulation (DESR) scale and WAIS scores with four subindex scores, in 418 adult patients diagnosed with ADHD in a psychiatric outpatient clinic specializing in the examination and treatment of ADHD

	All (*n* = 418)	Male (*n* = 230)	Female (*n* = 188)
Age range	18–69	18–67	18–69
Age: mean (*SD*)	36.6 (11.5)	36.1 (11.8)	37.2 (11.1)
Education years (%)			
≤12	203 (48.6)	122 (53.0)	81 (43.1)
13–15	179 (42.8)	92 (40.0)	87 (48.6)
>15	36 (8.6)	16 (7.0)	20 (10.6)
Married/cohabiting	188 (45.0)	105 (45.7)	83 (44.1)
Living with children	178 (42.6)	86 (37.4)	92 (48.9)*
Employment	258 (61.7)	149 (64.8)	109 (58.0)*
Depression‐MADRS score, mean (*SD*)	12.4 (6.4)	12.4 (6.9)	12.4 (5.7)
Comorbidity			
Depressive disorders (%)	54 (12.9)	28 (12.2)	26 (13.8)
Anxiety disorders (%)	133 (31.8)	58 (25.2)	75 (39.9)**
Substance use disorders (%)	57 (13.6)	36 (15.7)	21 (11.2)
ASRS: Attention deficit score, mean (*SD*)	27.3 (4.4)	26.9 (4.4)	27.8 (4.2)*
ASRS: Hyperactivity‐impulsivity score, mean (SD)	23.8 (6.5)	23.1 (6.7)	24.6 (6.3)*
Emotional dysregulation score, mean (SD)	12.1 (5.5)	11.0 (5.6)	13.4 (5.0)***
WAIS total score: FSIQ			
Mean (*SD*)	103.2 (13.8)	104.8 (13.5)	101.3 (14.0)**
Range	66–151	71–151	66–145
Verbal Comprehension Index			
Mean (*SD*)	105.9 (12.7)	107.2 (12.6)	104.2 (12.7)**
Range	74–161	82–150	74–145
Perceptual Organizational Index			
Mean (*SD*)	110.5 (16.0)	111.6 (15.8)	109.2 (16.2)
Range	70–150	70–150	72–148
Working Memory Index (WMI)			
Mean (*SD*)	90.5 (13.1)	92.3 (12.3)	88.2 (13.1)**
Range	57–136	65–136	57–126
Processing Speed Index (PSI)			
Mean (*SD*)	88.1 (12.5)	88.3 (12.7)	87.2 (12.3)
Range	57–125	60–125	57–120

Note

Numbers (percentage) or means (standard deviation) are reported. Sexes are compared with the chi‐squared test or *t*‐test.

* *p* < .05.

** *p* < .01.

*** *p* < .001. (Females compared with males.)

The mean hyperactive‐impulsive score (*SD*), mean attention deficit score (*SD*), and mean emotional dysregulation score (*SD*) are reported. Females in our sample scored higher on all three measures. The table also shows the WAIS profile of our sample. The mean full‐scale intelligence quotient (FSIQ) in our sample was 103.2 (*SD*: 13.8), and females had an average FSIQ of 101.3, while men had an average FSIQ of 104.8. Both sexes had typical profiles with significantly reduced scores on the Working Memory Index (WMI) and Processing Speed Index (PSI) compared to those expected based on their FSIQ. Significant correlations (*p* < .001) were found between all four subindex scores on the WAIS (data not shown).

The severity of attention deficits, as reflected in the ASRS attention deficit subscale score, was significantly correlated only with the PSI (Pearson correlation −.16, *p* < .001).

The severity of hyperactivity and impulsivity, as measured by the ASRS hyperactivity‐impulsivity subscale, was significantly correlated with the FSIQ (Pearson correlation −.19, *p* < .001) and all four subindex scores but was not associated with any of the independent variables in the adjusted model (data not shown).

The severity of emotional dysregulation, as measured by the DESR scale score, was significantly correlated with the FSIQ (Pearson correlation −.23, *p* < .001) and with all four subindex scores.

Table 2 shows the linear regression model of the associations between the outcome/dependent variable attention deficit severity, as measured by the ASRS inattention subscore, and the independent variables of age, sex, education level, depression, the WMI, and the PSI. The PSI (β = −.056, CI −0.094 to −0.017, *p* = .005) but not the WMI (β = −.012, CI: −0.049 to 0.025, *p* = .52) was associated with attention deficits. The *R* square (explained variance) for the fully adjusted model was .026, that is the model explained only 2.6% of the variance in attention deficits in our sample. None of the covariates were significantly associated.

**Table 2:** Associations between attention deficits measured by the ASRS inattention subscore as an outcome/dependent variable and age, sex, education level, depression measured by the MADRS score, Working Memory Index (WMI) and Processing Speed Index (PSI) as independent variables in a clinical sample of 418 adult ADHD patients. Linear regression

	Unadjusted				Adjusted			
	*B*	95% CI	*p*‐Value	*R* ^2^	*B*	95% CI	*p*‐Value	*R* ^2^
Age	.002	−0.039 to 0.034	.910	−.002	−.003	−0.039 to 0.034	.882	.026
Sex (male vs. female)	−.922	−1.76 to −0.082	.031	.009	−.781	−1.635 to 0.073	.073	
Education (3 level)	.164	−0.49 to 0.82	.621	−.002	.520	−0.188 to 1.229	.150	
Depression (MADRS score)	.003	−0.063 to 0.069	.929	−.002	−.001	−0.067 to 0.064	.965	
Working Memory Index	−.035	−0.067 to 0.003	.033	.009	−.012	−0.049 to 0.025	.524	
Processing Speed Index	−.054	−0.087 to −0.021	.002	.021	−.056	−0.094 to −0.017	.005	

Table 3 shows the linear regression model of the associations between the severity of emotional dysregulation, as measured by the DESR scale, as the outcome/dependent variable and the independent variables of age, sex, education level, depression, the WMI and the PSI. Neither the WMI (β = −.05, CI: −0.095 to −0.002, *p* = .041) nor the PSI (β = −.03, CI: −0.08 to −0.13 *p* = .22) were associated with the severity of emotional dysregulation in the adjusted model after the Bonferroni correction, with significance based on *p* < .025. Depression was associated with the severity of emotional dysregulation (β = .13, 95% CI: 0.05–0.21, *p* = .002). The *R*‐squared value (explained variance) for the fully adjusted model was .098, that is the model explained only 9.8% of the variance in emotional dysregulation in our sample. Age and education level did not contribute to the explained variance, but being female was significantly associated with emotional dysregulation (β = −2.13, 95% CI: −3.18 to −1.09, *p* < .001), and depression was also significantly associated with emotional dysregulation (β = .13, 95% CI: 0.05–0.21, *p* = .002).

**Table 3:** Associations between emotional dysregulation measured by the Deficient Emotional Self‐Regulation (DESR) scale as an outcome/dependent variable and age, sex, education level, depression (MADRS score), Working Memory Index (WMI) and Processing Speed Index (PSI) as independent variables in a clinical sample of 418 adult ADHD patients. Linear regression

	Unadjusted/crude				Adjusted			
	*B*	95% CI	*p*‐Value	*R* ^2^	*B*	95% CI	*p*‐Value	*R* ^2^
Age	.018	−0.03 to 0.06	.43	.001	.021	−0.02 to 0.066	.35	.098
Sex (male vs. female)	−2.36	−3.40 to −1.32	<.001	.046	−2.13	−3.18 to −1.09	<.001	
Education (3 level)	−.61	−1.44 to 0.22	.15	.005	−.16	−1.03 to 0.71	.72	
Depression (MADRS score)	.14	0.06 to 0.23	.001	.026	.13	0.05 to 0.21	.002	
Working Memory Index	−.08	−0.12 to −0.040	<.001	.035	−.05	−0.095 to −0.002	.041	
Processing Speed Index	−.058	−0.10 to −0.016	.007	.017	−.03	−0.08 to −0.13	.22	

Table 4 shows the nine attention deficit items in the DSM‐5 ADHD criteria and the nine corresponding inattentive items on the ASRS, the Pearson correlations with the WMI and PSI and the significance (2‐tailed). Four items pertaining to attention deficits were correlated with the WMI (ASRS items 1, 3, 7, and 9), and four items were correlated with the PSI (ASRS items 2, 3, 9, and 11). Three items were correlated with neither (ASRS items 4, 8, and 10).

**Table 4:** DSM‐5 ADHD criteria: nine items pertaining to attention deficits, the corresponding nine ASRS inattentive items and their correlations with the Working Memory Index (WMI) and Processing Speed Index (PSI) in a clinical sample of 418 adult patients with ADHD

DSM‐5: attention deficit items	ASRS Inattentive items	Working Memory Index (WMI)		Processing Speed Index (PSI)	
		Person correlation	Sign. (*p*)	Pearson correlation	Sign. (*p*)
**a.** Often fails to pay close attention to details or makes careless mistakes in schoolwork, at work or during other activities	**1.** How often do you have trouble wrapping up the final details of a project, once the challenging parts have been done?	.120	.014	−.002	.964
**b.** Often has difficulty sustaining attention in tasks or play activities	**8.** How often do you have difficulty keeping your attention when you are doing boring or repetitive work?	−.031	.527	−.020	.678
**c.** Often does not seem to listen when spoken to directly	**9.** How often do you have difficulty concentrating on what people say to you, even when they are speaking to you directly?	−.221	<.001	−.106	.030
**d.** Often does not follow through instructions and fails to finish schoolwork, chores, or duties in workplace	**7.** How often do you make careless mistakes when you have to work on a boring or difficult project?	−.141	.004	−.066	.177
**e.** Often has difficulty organizing tasks and activities	**2.** How often do you have difficulty getting things in order when you have to do a task that requires organization?	.009	.862	−.166	.001
**f.** Often avoids, dislikes, or is reluctant to engage in tasks that require sustained mental effort	**4.** When you have a task that requires a lot of thought, how often do you avoid or delay getting started?	.004	.939	−.087	.077
**g.** Often loses things necessary for tasks and activities	**10**. How often do you misplace or have difficulty finding things at home or at work?	−.036	.458	−.054	.267
**h.** Is often easily distracted by extraneous stimuli	**11**. How often are you distracted by activity or noise around you?	−.068	.162	−.099	.043
**i**. Is often forgetful in daily activities	**3.** How often do you have problems remembering appointments or obligations?	−.098	.044	−.141	.004

Note

Pearson correlations with significance (2‐tailed) for each item are shown.

Table 5 shows the eight items pertaining to emotional dysregulation composing the Deficient Emotional Self‐Regulation (DESR) scale, the Pearson correlations with the WMI and the PSI and the significance (2‐tailed). All but one item (DESR item 4: Easily excited by activities going on around me) were correlated with both the WMI and PSI.

**Table 5:** Emotional dysregulation was measured as Deficient Emotional Self‐Regulation (DESR) scale items and correlated with the working memory index (WMI) and processing speed index (PSI) in a clinical sample of 418 adult patients with ADHD

Deficient Emotional Self‐Regulation (DESR) items:	Working Memory Index (WMI)		Processing Speed Index (PSI)	
	Pearson correlation	Significance (*p*)	Pearson correlation	Significance (*p*)
1: Quick to get angry or become upset	−.188	<.001	−.112	.022
2: Easily frustrated	−.157	.001	−.126	.010
3: Overreact emotionally	−.139	<.001	−.145	.003
4: Easily excited by activities going on around me	−.015	.762	.057	.245
5: Lose my temper	−.209	<.001	−.111	.024
6: Argue with others	−.116	.018	−.142	.004
7: Am touchy or easily annoyed by others	−.113	.022	−.136	.006
8: Am angry or resentful	−.145	.003	−.101	.039

Note

Pearson correlations with significance (2‐tailed) for each item are shown.

## Discussion

The main finding in this study was that the correlations between the psychometric tests of verbal working memory and processing speed and the severity of attention deficits and emotional dysregulation were weak (*R* ^2^ < .1) and mostly not significant. Only processing speed was associated with the severity of attention deficits (β = −.056, *p* = .003). The psychometric WAIS test index scores for verbal working memory (WMI) and processing speed (PSI) seem to have limited utility as indicators of the severity of inattention and emotional dysregulation in adults with ADHD.

In our sample, 51.4% of the patients had attained a university degree or graduate degree, which is higher than the proportion of the general Norwegian population (34.6%; https://www.ssb.no/en/utdanning/statistikker/utniv). Participants in our sample had a 61.7% employment rate, which is lower than the 70.3% in the general population (https://www.ssb.no/en/arbeid‐og‐lonn/statistikker/aku).

In our sample, we found a mean FSIQ of 103.1 (Table 1), which is just above the general population mean, indicating good cognitive function. This finding is contrary to previous studies that have shown ADHD patients as a group to have lower FSIQ scores than the population‐based norms (Bridgett & Walker, 2006), primarily due to reduced scores on the WMI and PSI (Harrison, DeLisle, & Parker, 2008; Iverson, Lange, Viljoen, & Brink, 2006; Theiling & Petermann, 2014; Wechsler, Coalson, & Raiford, 2008). There has been a minor discussion regarding whether adults with ADHD perform differently from normal controls on the FSIQ (Faraone & Biederman, 2005; Nigg et al., 2005), but our findings confirmed a normal FSIQ mean score and range, at least in this sample.

We found significant reductions in the WMI and PSI scores compared to the FSIQ scores for both sexes (Table 1), which is in line with the findings reported in the literature (Harrison et al., 2008; Iverson et al., 2006; Theiling & Petermann, 2014; Wechsler et al., 2008).

In our sample, verbal working memory was not associated with the severity of attention deficits in the adjusted model. This is in line with other studies, which found only weak correlations between working memory scores and self‐reports in individuals with ADHD (Barkley & Murphy, 2011). Self‐reported symptom severity measures in adult ADHD patients usually do not correlate with the objective psychometric performance tests (Woods et al., 2002) and correlate only weakly with WAIS performance (Theiling, Petermann, & Daseking, 2013). Others have found that ADHD symptom severity correlated significantly with working memory (Alderson et al., 2013; Brydges, Ozolnieks, & Roberts, 2017), but the correlations between working memory tests and self‐reported rating scales were weak and mostly not significant (Barkley & Murphy, 2011). All these findings indicate that the importance of working memory deficits in ADHD is consistent and persists into adulthood and that methodological variability may explain why working memory deficits have not been uniformly detected (Alderson et al., 2013; Kasper, Alderson, & Hudec, 2012).

According to Baddeley’s definition of working memory, there are two modality‐specific components, namely a phonological loop (verbal working memory) and a visuospatial sketchpad (Baddeley et al., 1999). Earlier research has found attention deficits in ADHD to be more closely tied to the visuospatial component than to the phonological loop (Martinussen, Hayden, Hogg‐Johnson, & Tannock, 2005; Rapport et al., 2008; Rhodes, Park, Seth, & Coghill, 2012).

In our sample, the severity of attention deficits was not associated with verbal working memory (the phonological loop), as measured by the WMI, after adjusting for other covariates (Table 2). This is in line with previous studies that found that measures of attention deficits in ADHD are unrelated to verbal working memory (Martinussen et al., 2005); however, other studies found that verbal working memory is an important domain of cognitive dysfunction in ADHD (Ramos, Hamdan, & Machado, 2019).

In our sample, the WMI correlated with four out of nine inattentive items on the ASRS (see Table 4), which underscores the fact that the WMI captures only a part of the attention deficits in ADHD, namely, the ‘verbal working memory deficit’. Verbal working memory is commonly viewed as the temporary maintenance of verbal information and is an immediate form of memory used to transform verbal information, such as speech, into meaning (Caplan & Waters, 1999). A person with impaired verbal working memory might experience the following: ‘I hear what you say, but I don’t understand what you mean’. A specific question to assess this would be: *Do you often have difficulties understanding what people say? Do you often misinterpret what other people say? Do you often have difficulties interpreting the meaning of a sentence spoken to you? Do other people often comment that you have not understood their verbal intention or sentence?* These questions would enable us to better understand specific parts of the clinical difficulties involved in a verbal working memory deficit, and these suggested questions are slightly different from the four verbal attention deficit items in the DSM (See Table 4. ASRS item 1, 3, 7, 9).

In our sample, the severity of attention deficits was associated with decreased scores for processing speed, such as the PSI (see Table 2). This is in line with other studies in adults with ADHD (Barkley & Murphy, 2011; Schweiger, Abramovitch, Doniger, & Simon, 2007; Shanahan et al., 2006). An impaired PSI has been found to correlate with inattention in children with ADHD (Kubo et al., 2018; Thaler et al., 2013; Yang et al., 2013). Severe attention deficits in children correlate with an impaired PSI (Jacobson, Geist, & Mahone, 2018). Our finding is in line with other studies that have shown that adults with ADHD have significant reductions in processing speed, as reflected in low PSI scores on the WAIS‐IV (Wechsler et al., 2008). Other studies have not found that processing speed is associated with ADHD symptom severity (Brydges et al., 2017). Impaired processing speed has been reported to be weakly correlated with self‐reported attention deficit ratings given by individuals with ADHD (Barkley & Murphy, 2011).

In our sample, the PSI correlated with four out of nine items pertaining to attention deficits (ASRS scores for attention deficit, Table 4), which underscores that the PSI captures only a part of attention deficits in ADHD, namely the ‘processing speed deficit’. Processing speed is the pace at which you take in information, make sense of it, and begin to respond (Horning & Davis, 2012). In our sample, we found that the mean processing speed was significantly reduced compared to the mean FSIQ score. Questions that could solicit information about processing speed would be as follows: *How often do you have difficulties starting boring tasks you should have done? How often is it difficult to stay on the right track for your boring tasks? How often do you miss meeting the goal of your boring tasks?* These questions are also slightly different from the four original DSM items that correlated significantly with processing speed (see Table 4: ASRS items 2, 3, 9, 11).

In our study, emotional dysregulation was not associated with verbal working memory or the WMI score after adjusting for covariates (see Table 3). This is in contrast to a previous study that found that deficits in verbal working memory were associated with the severity of parent‐rated emotional dysregulation in children with ADHD (Uderman, 2015); however, other studies have not found this association in adults with ADHD (Gisbert et al., 2019; Surman et al., 2015). In our sample, both depression and female sex were significantly associated with emotional dysregulation and may to some extents have affected the adjusted regression analysis. Additionally, applying the Bonferroni correction and considering differences significant if *p* < .025 favoured a non‐significant result. In an unadjusted model, emotional dysregulation was associated with both the WMI (*B* = −.08, 95% CI: −0.12 to −0.040, *p* < .001, *R* ^2^ = .035) and PSI (*B* = −.058, 95% CI: −0.10 to −0.016 *p* = .007, *R* ^2^ = .017). There were correlations between emotional dysregulation and verbal working memory and processing speed, but the causal relations were difficult to assess.

Verbal working memory impairment has long been recognized as an important domain of cognitive dysfunction in ADHD (Moore et al., 2006), and since verbal working memory tasks typically involve the ability to recall language perception and production (Acheson & MacDonald, 2009), decreased scores for verbal working memory may reflect impulsive and aggressive verbal behaviour (Kockler & Stanford, 2008). Our finding underscores these earlier findings that aggression, as defined in emotional dysregulation, is linked to verbal working memory deficits.

Decreased executive function has long been linked to physical aggression in boys (Séguin, Pihl, Harden, Tremblay, & Boulerice, 1995), and poor verbal working memory storage in children may be associated with greater peer rejection (McQuade, Murray‐Close, Shoulberg, & Hoza, 2013). The association between higher levels of aggression and decreased levels of verbal working memory has also been seen in studies in the general adult population (Colby, 2008), and this may provide a new goal for verbal working memory training, that is, a reduction in emotional dysregulation. People with low verbal working memory capacity compared with their FSIQ score may not comprehend all relevant arguments in a verbal discussion and may therefore misunderstand, misinterpret, or misjudge more easily. Misunderstanding is a main source of anger (Lochman, Barry, Powell, & Young, 2010). If so, this may encourage clinicians to try verbal working memory training as an additional therapeutic approach to achieve better emotional regulation in ADHD patients, although anger regulation and other aspects of emotional dysregulation should still be treated directly.

In our sample, the emotional dysregulation items on the DESR scale correlated with both verbal working memory and processing speed, with correlations observed for seven out of the eight items on the DESR scale (see Table 5). The relationship between anger and cognitive distortions (Chereji et al., 2012) and the frequencies of both the expression of anger and cognitive distortion have been linked to decreased executive functioning (Persampiere et al., 2014). Explosive anger, labelled emotional dysregulation, has been understood as a distortion and a disabling feature of ADHD (Bunford et al., 2018; Skirrow & Asherson, 2013).

### Methodological considerations

In this paper, we assumed that the participants reported their true beliefs regarding their subjective dysfunction. This clinical report from the patient is the basis for the diagnosis of ADHD, and in our paper, for the measurements of the severity of attention deficits and emotional dysregulation. Patients may, however, underreport or exaggerate their difficulties. What is considered to be the reference state of normality may also differ. In our paper, we considered what the patient reported to be fact, even though it was a subjective truth.

Psychometric tests are more objective. However, the results may not reflect everyday functioning. It is possible that some patients score well in a controlled, optimized test situation but are more impaired in their everyday life. This may result in clients reporting severe attention deficits in everyday life without having impaired test scores. When these differences between subjective reports and objective test results occur, they are not necessarily conflicting but may rather complement each other.

The sex differences we see in Table 1 reflect the participants in our sample. There is a selection bias given that the two sexes may seek help at different levels of impairment, and therefore, we cannot generalize sex differences in our sample to the general ADHD population.

Despite the performance of extensive psychological, biological, and neuroscientific studies, it has not been possible to establish a general unified agreement on the categorization of working memory; therefore, several working memory tasks are used in working memory studies (such as the Sternberg task, n‐back task, Corsi block‐tapping test, Wechsler’s Memory Scale, and working memory subtests of the WAIS; Chai, Abd Hamid, & Abdullah, 2018). Barkley suggested that working memory is one out of four executive functions that is impaired in ADHD (Barkley, 1997) and that this could be assessed by a questionnaire such as the Behaviour Rating Inventory of Executive Function (BRIEF; Gioia, Isquith, Guy, & Kenworthy, 2000). Even though the WAIS is the most frequently used psychometric test among clinical neuropsychologists (Rabin, Paolillo, & Barr, 2016) and there is a consensus that the WAIS measures working memory to some extent (Hill et al., 2010), it has certain limitations with regard to measuring working memory. There are three reasons to be wary of using the WAIS WMI to measure working memory:

The first reason is that the WMI only measures verbal and not visuospatial working memory, so the question is whether the WMI measures not the true ‘working memory’ of a person but rather their verbal understanding and education (Chai et al., 2018). The tests are only auditory. Tests of visual working memory that could have completed the picture are lacking (Egeland, 2015; Egeland, Bosnes, & Johansen, 2008). However, according to Baddeley (Baddeley & Hitch, 2001; Baddeley et al., 1974), the most important part of working memory is the central executive function, which is thought to not be modality‐specific.

The second reason is that the WMI includes an arithmetic task that also correlates with verbal comprehension tests because linguistically formulated assignments place substantial demands on verbal understanding (Arnau & Thompson, 2000). The correlation between arithmetic and language tests may also be due to a third variable. Good linguistic and technical accounting skills are particularly affected by education, and calculation tasks can hardly be considered working memory tests (Egeland et al., 2008).

The third reason is that reduced scores on the WMI are not specific for ADHD but are sensitive for nearly all psychiatric disorders, while the PSI is more specific for ADHD (Egeland et al., 2009; Theiling et al., 2013). Others have explained this based on the fact that WMI impairment may be related to symptom severity in general psychopathology (Brydges et al., 2017).

In our sample, we had a wide range of FSIQ estimates. It is possible that participants with a very high FSIQ will experience severe problems even when they have a moderate index score of WMI or PSI within the normal range. Adjustments for this were not performed in this paper.

### Strengths and limitations

The strengths of the study include a naturalistic design with the inclusion of a large number of participants referred for the examination and treatment of ADHD. They were recruited from a large area and over a long period of time. There were no exclusion criteria, and everyone who met the ADHD criteria was asked to participate in the study. The rate of consent and the number of participants were high.

The inclusion of patients attending a private and not a governmentally funded ADHD clinic questions the representativeness of the study sample with regard to the adult population with ADHD in general. The participants may have a higher socioeconomic status and be less impaired than those attending public outpatient clinics or hospitals. Additionally, the prevalence of comorbidities may not be representative of those in the total ADHD patient population. However, we assume that differences in the sample selection may primarily affect the mean scores and, to a lesser extent, the associations between the mean scores (Nohr & Olsen, 2013; Rothman, Gallacher, & Hatch, 2013). The associations between WAIS scores and ADHD or emotional dysregulation severity should therefore be considered in a more general context.

The use of the WAIS‐III and not the newer WAIS‐IV is a weakness of this study. The WAIS‐IV provides superior measurement, scoring, and structural models to measure the FSIQ than the WAIS‐III (And & Benson, 2013), but working memory is still tested only in the auditory modality (Egeland, 2015). Both the WAIS‐III and WAIS‐IV detect reductions in working memory and processing speed in adults with ADHD, which suggests that the identification of such deficits with both versions of the WAIS remains robust (Wechsler et al., 2008). Altogether, the WAIS‐IV is better and newer but not a radically different assessment instrument than the WAIS‐III. We therefore believe the statistical results and the results reported in this article would not have been radically different if the WAIS‐IV had been used.

### Clinical implications

The symptoms of attention deficits in ADHD are related to the subjective experience of being inattentive and are associated with the objective finding of processing speed deficits. Emotional dysregulation is a subjective awareness of experiencing explosive anger but was not associated with verbal working memory (WMI) or processing speed (PSI) as measured by the WAIS test.

In our adult ADHD sample, we found typical reductions in the WMI and PSI, but only the PSI was associated with attention deficits, and the power of this association was limited. It seems that objectively tested verbal working memory and processing speed measure only parts of the subjective experience of attention deficits and emotional dysregulation.

Extending the WAIS test battery by including measures of visuospatial working memory could make this psychometric tool more suitable for measuring attention deficits in ADHD.

Weak correlations between items of attention deficit (ASRS subscore) and emotional dysregulation (DESR) indicate that the questions on these questionnaires do not fully reflect verbal working memory and processing speed. Alternative questions, as suggested in the paper, may better reflect these parameters.

### Conclusions

The participants in our adult ADHD sample had normal mean FSIQ score, with the expected typical reduction in verbal working memory, as reflected on the WMI, and processing speed, as reflected on the PSI. Only processing speed was associated with the severity of attention deficits, but the effect size of the association was small. Our results indicate that the correlations between the severity of attention deficits and emotional dysregulation and psychometric tests of verbal working memory and processing speed were weak (*R* ^2^ < .1) and mostly not significant. The psychometric WAIS WMI and PSI seem to have limited utility as indicators of the severity of attention deficits and emotional dysregulation in adult ADHD patients.

## Conflicts of interest

EA has received speaker honoraria from Shire, TH and GO report no conflicts of interest.

## Authors’ contributions

EA and TH designed the study. EA collected and analysed the data. EA and TH actively participated in the writing of the manuscript and all authors approved the final draft. GØ commented on the manuscript draft and suggested changes.

## Ethical approval and consent to participate

The study was approved by the Regional Medical Ethics Committee, South‐East D, Norway, 2015/426. Written consent to participate was obtained from all participants.

## Consent for publication

Not applicable.

## Declarations

We confirm that all methods were performed in accordance with relevant guidelines and regulations.

## Data Availability

Data are from a private psychiatric outpatient clinic in Oslo. Public availability would compromise privacy of the respondents. According to the approval from the Norwegian Regional committees for medical and health research ethics, the data are to be stored properly and in line with the Norwegian Law of privacy protection. However, anonymized data are freely available to interested researchers upon request, pending ethical approval from the ethics committee. Interested researchers can contact project leader Espen Anker (espen.anker@online.no) with requests for the data.
